# Development of a set of value-based healthcare preconditions supporting military trauma patients in military operations: a Delphi study

**DOI:** 10.1136/bmjopen-2025-101224

**Published:** 2025-12-19

**Authors:** Henk van der Wal, Tonnie Dulk, Thijs van Dongen, Rigo Hoencamp, Kees Ahaus

**Affiliations:** 1 Department of Surgery, Trauma Research Unit, Erasmus MC, University Medical Centre Rotterdam, Rotterdam, the Netherlands; 2 Defense Healthcare Organisation, Netherlands Ministry of Defence, Utrecht, the Netherlands; 3 Department of Surgery, Alrijne Hospital Leiden, Leiden, the Netherlands; 4 Erasmus School of Health Policy and Management, Department Health Services Management and Organisation, Erasmus University Rotterdam, Rotterdam, the Netherlands

**Keywords:** Organisation of health services, Quality in health care, Patient-Centered Care, Patient Reported Outcome Measures, Surveys and Questionnaires, Trauma

## Abstract

**Abstract:**

**Objectives:**

To explore the perceived importance of essential Value-Based Healthcare (VBHC) and patient-centred care elements within operational military healthcare among wounded service members (WSM), military surgeons (MS) and medical commanders, and to identify preconditions that enable the delivery of patient-centred care and relevant medical outcomes during military operations.

**Design:**

A two-round Delphi study was conducted following the Accurate Consensus Reporting Document guideline. An initial list of 17 preconditions was developed through a narrative-style literature review and expert-panel discussion. Preconditions were rated on a four-point Likert scale (‘not important’ to ‘very important’) to reach consensus on ‘military-oriented’ preconditions for patient-centred trauma care.

**Setting:**

An in-person expert-panel meeting and subsequent online Delphi surveys conducted between March 2020 and September 2022.

**Participants:**

The expert panel consisted of Dutch military healthcare leaders and clinical specialists. A total of 30 participants completed both Delphi rounds, including 17 MS and commanding officers (Group 1) and 13 WSM (Group 2) with deployment experience in Uruzgan, Afghanistan, ensuring balanced representation of care providers, facilitators and recipients.

**Outcome measures:**

Primary outcome: identification of military-oriented preconditions essential for patient-centred and value-based trauma care. Secondary outcome: conceptual contribution to the future development of patient-centred outcome measures for military trauma populations.

**Results:**

Consensus was reached on 10 preconditions perceived as important or very important. While Group 1 prioritised operational readiness and procedural efficiency, Group 2 emphasised communication, shared decision-making and family involvement. Both groups rated informed consent, timely treatment registration and patient safety as the most critical elements, demonstrating convergence between logistic-oriented and patient-oriented perspectives.

**Conclusions:**

Integrating VBHC principles into military medical doctrine can reconcile operational readiness with patient-centred care. The consensus-based preconditions identified in this study provide a foundation for developing measurable outcomes that reflect value for both patients and the military health system and can guide future VBHC implementation in trauma-related operational care.

**STRENGTHS AND LIMITATIONS OF THIS STUDY**

- The Delphi method was applied in accordance with the Accurate Consensus Reporting Document reporting guideline to ensure transparency and reproducibility.
- Two stakeholder groups—care providers/facilitators and care recipients—were included to balance perspectives within the consensus process.
- A narrative-style literature review combined with expert-panel input guided the formulation of 17 preconditions in a field lacking standardised terminology.
- Anonymous Delphi rounds reduced potential hierarchical or dominant influence on consensus outcomes.
- All participants were recruited from Dutch military healthcare, which may limit the generalisability of the findings to other international contexts.

## Introduction

 The 2023 North Atlantic Treaty Organisation (NATO) summit in Vilnius reaffirmed the need to strengthen collective defence and resilience in an increasingly complex security environment.^1^ Aligned with this, the 2022 Netherlands Defence White Paper prioritised reinforcing the Dutch Armed Forces, including logistics, capabilities and the military medical support chain.^2^ Dutch military medicine has long been grounded in civil-military integration, embedding clinical practice within civilian healthcare and mutually sharing operational and clinical lessons to enhance both systems.^3^ In today’s evolving geopolitical context, stronger collaboration is essential to access and integrate rapid civilian healthcare innovations. NATO’s ‘Continuum of Care’ model structures military healthcare across deployed and home-base capabilities.^4 5^ Although doctrine defines it as patient-centred, its operational reality remains largely output-oriented—focused on readiness, throughput and treatment capacity—rather than outcome-oriented, prioritising long-term recovery, functionality and quality of life. Nonetheless, there is a clear relationship between the health outcomes of individual service members and the readiness of the medical system. Contributing to good health outcomes can therefore be seen as a precondition of the medical system for military operational readiness.

The concept of Value-Based Healthcare (VBHC), introduced by Porter and Teisberg in 2006,^6^ provides a compelling framework for addressing this imbalance. VBHC defines value as the achieved health outcomes that matter most to patients relative to the costs required to achieve them,^7^ advocating a transformation from volume-based to outcome-based healthcare. Its implementation requires organising delivery of care around patient needs, measuring outcomes across the full cycle of care, and using these data to continuously improve quality and efficiency. While several countries have initiated large-scale VBHC transformations,^8 9^ its integration into military healthcare systems remains limited. The operationalisation of the overarching strategy has been shaped by the ‘Value Agenda’.^10^ Recent progress in outcome standardisation—such as the International Consortium for Health Outcomes Measurement (ICHOM) ‘Set of Patient-Centred Outcome Measures for Patients with Major Injury’ published in 2025—creates new opportunities for application in military trauma care.^11^ Exploring how VBHC principles and patient-centred outcome measurement can be embedded within military trauma systems may offer a new pathway to enhance both patient recovery and mission readiness, aligning medical effectiveness with the strategic imperatives of modern military medicine. Within the operational medical chain, healthcare professionals determine what is clinically feasible based on established clinical practice guidelines^12^ and available resources, while medical treatment facility (MTF) commanders define what is logistically and operationally achievable. Ensuring quality and safety therefore requires balancing medical feasibility with operational sustainability.^13^ Although this system depends heavily on logistics and limits patient choice, civilian healthcare trends increasingly emphasise patient-centred and participatory approaches,^14^ which remain challenging yet important to adapt within military health systems.

We aimed to explore how important essential VBHC and patient-centred care elements related to operational military healthcare are perceived by wounded service members (WSM), military surgeons (MS) and military medical commanders (MCO). Our goal was to identify preconditions—defined as the essential medical, logistical, operational and contextual factors that must be in place to enable the delivery of patient-centred care and VBHC within the military operational environment—for the preferred and relevant medical outcomes for military patients with acute trauma during military operations. In this context, preconditions encompass the balance between what is clinically feasible (as determined by healthcare professionals following medical guidelines and available resources) and what is logistically and operationally possible (as determined by military leadership). These elements represent the fundamental requirements that determine the potential for achieving desired medical outcomes and patient-centred approaches during military operations. To address this, we conducted the following research question: for what preconditions can consensus be reached regarding their importance in delivering patient-centred care and medical outcomes during military operations?

## Methods

### Design and setting

The origin of this study was the necessity to find preconditions for the preferred and relevant medical outcome for the WSM during military operations. A Delphi method was used to conduct the study to reach consensus where discrepancies exist and empirical evidence is absent.^15^,^17^ The Accurate Consensus Reporting Document guideline^18^ for the use of Delphi processes in biomedical research was used as the basis for the consensus process, to improve the completeness and transparency of reporting (see online supplemental table 1).

### Participants

For participation in the study, we selected a sample from the deployment period of the Dutch Armed Forces in Uruzgan, Afghanistan, in the period 2006–2010.^19 20^ The participants in the study consisted of two groups: Group 1 (divided into two subgroups): (a) MS and (b) MCO of the Dutch MTF in Uruzgan; and Group 2: WSM formally categorised as Wounded in Action, (battle casualties^21^ ;). Although mental and physical trauma are indispensably related, this study focuses on military patients with physical trauma injuries. Prior to the invitation of the participants, the objective was to attain a balanced representation between Group 1 and Group 2 participants, while also ensuring an appropriate mix of experience across both groups.

### Delphi method

The Delphi method^22^ was used to reach consensus on the preconditions that could be included as ‘military-oriented’ and/or ‘clinical-oriented’. All statements were presented as preconditions and to be interpreted as normative statements—that is, as conditions that must or should be in place to enable patient-centred and value-based trauma care within military operations, even when such wording was not explicitly used. To formulate the preconditions, we set up an expert panel prior to conducting the Delphi survey, consisting of subject matter experts consisting of military healthcare leaders and clinical specialists, some with similar experience as the respondents. To compile the initial list of preconditions, we conducted a narrative style literature review^23 24^ of the (inter)national most relevant literature on VBHC and patient-centred care^710 25^,^30^ and literature and doctrines on operational military healthcare.^19^,^2131^ We used a narrative style review to support the expert panel interpretations, as the field of research on this topic is largely unexplored and lacks the associated standardised terminology. This list was discussed and validated by the expert panel and resulted in a definitive list of 17 preconditions; see online supplemental table 2. The expert panel members did not participate in the Delphi survey. The Delphi method ensures that preconditions can be scored anonymously, whereby opinions of more dominant personalities do not lead to undesirable influence.^38^ The respondents completed questionnaires in a two-round modified Delphi survey, scoring each precondition. The distribution was made on a four-point Likert scale: (a) ‘not important’, (b) ‘somewhat important’, (c) ‘important’ and (d) ‘very important’.^39 40^ After scoring the precondition, the respondent could also nominate the precondition for reformulation. The respondent could also propose an additional precondition for the next Delphi round. All surveys took place from March 2020 until September 2022. In line with other Delphi studies,^41^ preconditions scoring ‘important’ and ‘very important’ by at least 80% of the respondents were included as important or very important preconditions. When the scores of the respondents were between 50% and 80% for ‘important’ and ‘very important’, the precondition was resubmitted, with or without redrafting where applicable, in the second round. Scores above 50% for ‘not important’ and ‘somewhat important’ were excluded. This Delphi study, by reaching consensus on the preconditions presented in two rounds, resulted in a set of ‘military-oriented’ preconditions.

### Ranking of the preconditions

The included preconditions are presented in tables 4 and 5. The preconditions are rank ordered, first by mean (*x̅*), second by SD(s). The mean (*x̅*) indicates the average score of the precondition (ie, its perceived importance) according to our participants (rated by each participant on a four-point Likert scale). A precondition’s SD(s) was primarily used to rank order preconditions with a similar mean. In the case of preconditions with similar mean importance scores, their SDs were used as a secondary ranking criterion, with lower SDs indicating greater consensus among participants and therefore resulting in a higher rank. These tables also display whether preconditions were included in round 1 or 2.

### Patient and public involvement

A delegation of WSM was involved in defining the research question and design of the study. The delegation recruited all participants for the group of WSM, helping the researchers to appropriately engage these participants in the Delphi survey rounds. In agreement with the delegation, the participants were not actively involved in the further development of the paper. All participants will be informed via e-mail after publication of the paper. The first author has offered, if desired, to come and present the findings of the study at a location and time to be provided by the delegation.

## Results

Table 1 shows the participation of the respondents in our Delphi study. The demographic characteristics of the participants are summarised in table 2.

**Table 1:** Respondents’ survey—rounds 1 and 2

Response	Round 1			Round 2		
	Recruited (N)	Participated (N)	Participated (%)	Recruited (N)	Participated (N)	Participated (%)
Group 1						
MCO	11	7	64%	11	9	82%
MS	14	8	57%	14	8	57%
Group 2						
WSM	18	14	78%	18	13	72%
Total	43	29	67%	43	30	70%

MCO, Military commanding officers; MS, Military surgeons; WSM, Wounded service members.

**Table 2:** Characteristics of the participants

Characteristics	Category	Group 1: (a) MS and (b) MCO (n=17)	Group 2: WSM (n=13)
Gender	Male/female	76.5 %/23.5%	84.6 %/15.4%
Age (years)	Min–max	35–76	33–45
	Mean (SD)	52 (11.08)	38 (3.44)
Experience (years)	Min–max	9–32	8–26
	Mean (SD)	20 (6.43)	17 (5.05)
Deployments (no.)	Mean (SD)	6 (5.13)	2 (1.64)

MCO, Military commanding officers; MS, Military surgeons; WSM, Wounded service members.

Table 3 shows the results of the survey for both groups. Group 1 included nine preconditions in the first round and excluded two preconditions. Six preconditions were assessed in the second round. Although five preconditions were reformulated, the survey led to only one additional included precondition. See table 4 for the Group 1 included preconditions for both round 1 and 2. Group 2 included seven preconditions in the first round and excluded one precondition. Nine preconditions were assessed in the second round, without any reformulation or new suggestion, which led to five additional included preconditions. In the second survey round, no new preconditions or reformulations were suggested. Therefore, we assume saturation was reached. See table 5 for the Group 2 included preconditions for both round 1 and 2. Five preconditions, numbers 1, 4, 12, 15 and 16, were after two rounds definitively excluded by the groups.

**Table 3:** Results survey rounds 1 and 2

Results survey rounds 1 and 2								
	Group 1 (MS and MCO)				Group 2 (WSM)			
Response	Round 1		Round 2		Round 1		Round 2	
*Number of preconditions*	17	100%	6	100%	17	100%	9	100%
Consensus reached:								
*Included*	9	53%	1	17%	7	41%	5	56%
*Excluded*	2	12%	5	83%	1	6%	4	44%
Discordance:								
Unchanged	1	6%	0	0%	9	53%	0	0%
Reformulated	5	29%	0	0%	0	0%	0	0%
New suggested	0	0%	0	0%	0	0%	0	0%

MCO, Military commanding officers; MS, Military surgeons; WSM, Wounded service members.

**Table 4:** Group 1 included preconditions on perceived importance

Ranking	Precondition no.	Round	Included preconditions on perceived importance	Mean	SD
1	11	1	Delay of care in the deployment area must be kept to a minimum in view of its effect on medical outcomes	3.60	0.51
2	10	1	Patient safety must not be compromised	3.60	0.63
3	14	1	The registration of the treatment was carried out on time from entry to discharge, which led to a complete patient record	3.53	0.52
4	2	1	There is an ‘informed consent’ between patient and professional	3.47	0.64
5	7	1	The patient is involved in the time-out procedure at the OR	3.29	0.73
6	3	1	During the preparation (the mission preparation training) for the deployment, the ‘procedure in the event of injury’ was known in R2 MTF in Uruzgan	3.27	0.46
7	6	1	During the period of deployment at the R2 MTF, there were recognisable moments of consultation between the patient and the professional to discuss the treatment process together, known as SDM	3.20	0.68
8	9	1	Sufficient information must be available when reporting injuries (NATO 9-liner) to the R2 MTF	3.20	0.68
9	8	1	By placing more emphasis on consultation and making agreements, by the actors in the medical chain, added value is created for the treatment of the patient and his/her outcome	3.00	0.76
10	13	2	Based on the number of patients, it is possible to work event-driven, in which case the established procedures weren’t leading, but the circumstances regarding the wounded soldiers	2.82	0.53

All items are presented as preconditions and are to be read as normative statements describing conditions that must or should be in place.

9-liner, a nine-line message format for requesting medical evacuation (MEDEVAC) in military operations; MTF, Medical treatment facility; NATO, North Atlantic Treaty Organisation; OR, Operating room; R2 MTF, A Role 2 Medical Treatment Facility is a forward-deployed, modular field hospital providing damage control resuscitation and surgery; SDM, Shared decision making.

**Table 5:** Group 2 included preconditions on perceived importance

Ranking	Precondition no.	Round	Included preconditions on perceived importance	Mean	SD
1	13	2	Based on the number of patients, it is possible to work event-driven, in which case the established procedures weren’t leading, but the circumstances regarding the wounded soldiers	3.77	0.44
2	2	1	There is an ‘informed consent’ between patient and practitioner	3.71	0.47
3	17	2	Depending on the severity of the injury, the military personnel’s family must be explicitly involved in any treatment programme	3.54	0.97
4	14	1	The registration of the treatment was carried out on time from entry to discharge, which led to a complete patient record	3.50	0.52
5	10	1	Patient safety must not be compromised	3.43	0.76
6	7	2	The patient is involved in the time-out procedure at the OR	3.38	0.65
7	3	2	During the preparation (the mission preparation training) for the deployment, the ‘procedure in the event of injury’ was known in R2 MTF in Uruzgan	3.31	0.75
8	8	2	By placing more emphasis on consultation and making agreements by the actors in the medical chain, added value is created for the treatment of the patient and his/her outcome	3.31	0.75
9	6	1	During the period of deployment at the R2 MTF, there were recognisable moments of consultation between the patient and the practitioner to discuss the treatment process together, known as SDM	3.29	0.73
10	9	1	Sufficient information must be available when reporting injuries (NATO 9-liner) to the R2 MTF	3.21	0.70
11	11	1	Delay of care in the deployment area must be kept to a minimum in view of its effect on medical outcomes	3.14	0.66
12	5	1	All of the wounded soldiers’ wishes for treatment under special circumstances (treatment wishes) were stated in the medical file	3.07	0.83

All items are presented as preconditions and are to be read as normative statements describing conditions that must or should be in place.

9-liner, a nine-line message format for requesting medical evacuation (MEDEVAC) in military operations; MTF, Medical treatment facility; NATO, North Atlantic Treaty Organisation; OR, Operating room; R2 MTF, A Role 2 Medical Treatment Facility is a forward-deployed, modular field hospital providing damage control resuscitation and surgery; SDM, Shared decision making.

The assessment of reformulation quality and quantity showed distinct patterns between the groups. Group 1 provided an average of 6.8 suggestions per precondition, with contributions distributed between MS (4.2) and MCO (2.2). In contrast, Group 2 offered minimal input, averaging only 0.4 suggestions per precondition, and mainly noted that the wording could be made less complex.

Analysis of the qualitative comments revealed clear thematic variation across participants. The MS tended to emphasise patient-related aspects, treatment processes and legal or regulatory considerations, while the MCO focused more on procedural workflows and the operational feasibility of implementing the preconditions. This divergence reflected broader differences in knowledge, focus and expectations between the two groups. The most pronounced contrast in comment frequency occurred between the MS (61.7%, n=115) and WSM (6.1%, n=115). The MS primarily addressed organisational and clinically applicable aspects, whereas the WSM focused on individual preferences and perceived desirability. Finally, the WSM commented mainly on aspects of ‘communication with the patient’ or ‘no communication possible when unconscious’. For an overview of the comments, see online supplemental table 3.

**Figure 1:**
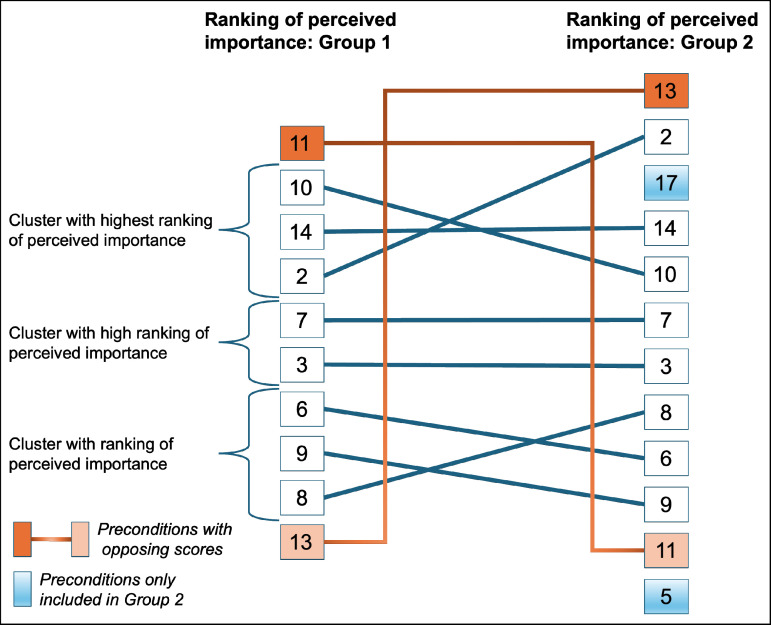
Comparison groups 1 and 2 on perceived importance. Legend: overview of the ranking and relationships in the perceived importance of the preconditions between groups 1 and 2.

Tables4  5 show, respectively, the 10 and 12 included preconditions in groups 1 and 2, based on the consensus reached. Overall, consensus between groups 1 and 2 was reached on 10 preconditions that are perceived as important. Figure 1 shows the relationships in the level of perceived importance of the preconditions between groups 1 and 2. Preconditions 2 ‘There is an ‘informed consent’ between patient and practitioner’, 10 ‘Patient safety must not be compromised’ and 14 ‘The registration of the treatment was carried out on time from entry to discharge, which led to a complete patient record’ scored as most important by both groups. Furthermore, in Group 2, two additional preconditions (5 ‘All of the wounded soldier’s wishes for treatment under special circumstances (treatment wishes) were stated in the medical file’ and 17 ‘Depending on the severity of the injury, the military personnel’s family must be explicitly involved in any treatment programme’) were included compared with Group 1, explicitly addressing the wishes of the WSM. Next, precondition 11 ‘Delay of care in the deployment area must be kept to a minimum in view of its effect on medical outcomes’. And precondition 13 ‘Based on the number of patients, it is possible to work event-driven, in which case the established procedures weren’t leading, but the circumstances regarding the wounded soldiers’ scored with the highest importance within the respective groups, reflecting respectively the highest priorities for each group, illustrating a contrast between organisational efficiency and patient-centred relevance.

Finally, it can be observed that the remaining eight preconditions to a large extent follow a similar ranking. With preconditions 2, 10 and 14, both groups show that in the continuum of care process, the patient should be the primary focus, preconditions 3 ‘During the preparation (the mission preparation training) for the deployment, the ‘procedure in the event of injury’ was known in R2 MTF in Uruzgan’. and 7 ‘The patient is involved in the time-out procedure at the OR’ focus on communicating with and involving the patient, and lastly, preconditions 6 ‘During the period of deployment at the R2 MTF saw recognisable moments of consultation between the patient and the practitioner to discuss the treatment process together, known as SDM’, 8 ‘By placing more emphasis on consultation and making agreements, by the actors in the medical chain, added value is created for the treatment of the patient and his/her outcome’ and 9 ‘Sufficient information must be available when reporting injuries. (NATO 9-liner) to the R2 MTF’ is also seen as important with communication as the guiding orientation. For further clarification, see online supplemental table 4.

## Discussion

This study aimed to increase the knowledge and understanding of preconditions for the preferred and relevant medical outcomes for a military acute trauma patient during military operations. This became evident through the differences in the respective military and civilian cycles of care, with the first being logistics-oriented and the second patient-oriented. Our Delphi study reached consensus among the participants regarding the identification of preconditions that can contribute to the delivery of patient-centred care and the development of medical outcomes in (military) trauma. Consensus between MS, MCO of the Dutch MTF in Uruzgan and WSM was reached on 10 preconditions.

### Target group observations

It was observed that there was a significant diversity in knowledge and expectations among the two groups. The diversity was reflected in various ways in the comments made in the rephrasing/comment boxes. Participants emphatically expressed their desire to share expectations. The rephrasing fields were predominantly used for personal reflection rather than actual reformulation, indicating that participants mainly used this space to contextualise or interpret the meaning of the preconditions rather than to propose alternative phrasing. The differences in the comments may partly reflect variations in age, military experience and deployment history, as well as the differing perspectives of patients, healthcare professionals and commanding officers, each engaging with the care process from distinct vantage points. In conclusion, despite some variation in perspectives, both groups showed considerable alignment, reaching consensus on most preconditions, suggesting that convergence outweighed divergence in their overall appraisal.^42^ Mutual respect and trust, from joint training, discipline and mentorship, and proficiency in performing the tasks, contribute to service members from all levels wanting to accomplish the mission in a goal-oriented way.^43^ The consensus reached may largely find its origin here.

### Importance of included preconditions

The included preconditions showed a perceived level of importance in both groups 1 and 2. Experience and literature confirm that rapid action in acute care is crucial^32^ and must be pursued in the right place and right time.^44^ This is to be seen from a clinical-reported outcome and not a patient-reported outcome. De WSM indicated that factors such as delay, routing and speed were of secondary concern when adequate escorting—preferably in their own language—was present. This underlines the difference in orientation between Group 1 (‘logistics-oriented’) and Group 2 (‘patient-oriented’). The findings highlight that while professional orientation shapes perceived importance, convergence exists around the fundamental principles of patient safety, documentation, communication and readiness. The highest-scoring preconditions (2, 10 and 14) particularly underscored the interrelationship between the WSM, MS and MCO. These reflect the need for informed consent between patient and healthcare provider,^45 46^ timely registration of all treatments as a prerequisite for good care,^10^ and the principle that patient safety is paramount and should not be compromised.^47^ These results suggest that for group 1, the integration of VBHC concepts into military medical doctrine may reconcile the apparent tension between operational readiness and patient-centred care. While event-driven flexibility^48^ allows adaptation to field circumstances, strict adherence to established procedures remains essential in large-scale collective defence scenarios such as NATO Article 5 operations,^49^ to maintain treatment capacity and standardisation. Group 2, on the other hand, perceives this more as the opportunity to provide emphatically patient-centred care.

The five preconditions perceived as highly important (7, 3, 6, 8 and 9) encompassed elements essential to both military medical chain design and patient-centred care, reflecting clear agreement between the groups’ logistic-oriented and patient-oriented perspectives. The time-out procedure (7) represents a key patient-safety measure^50^ directly associated with improved outcomes,^51^ while involving the service member and their family (3, 17) was considered crucial, as supporting preparation for a medical procedure^52^ parallels mission readiness. Regular consultation moments during deployment (6) illustrated shared decision-making^27^ and the establishment of international medical networks (8) that align with the VBHC principle of expanding excellent services across geography’,^10^ enhancing quality within the military medical evacuation chain.^4^ Overall, the convergence of both groups around shared decision-making, preparation and patient safety suggests that VBHC can strengthen outcomes and trust within the medical chain, with explicit involvement of service members and their families, continuous data registration and international collaboration serving as key enablers of both logistical efficiency and human-centred resilience within enhanced MHS.

### Measuring outcome

The value of standardised sets of patient-centred outcome measures, as designed and published by ICHOM, is recognised internationally.^53^ Such a set dedicated to the population of military patients who have an increased risk of major injury during deployment does not yet exist and will certainly have an added value. Gaining more insight into possible risks for the level of treatment with limited resources during mission preparations creates opportunities to make better choices around a treatment.^54^ This research helps by filling the above-mentioned gap and creating opportunities by focusing on the relevant preconditions for the development of patient-oriented and clinical-oriented outcomes for military patients with a traumatic injury during a mission, but is most likely also applicable to civilian patients in the trauma care domain.

### Limitations

The data collection, carried out in 2020/2022, covers the participants’ experiences of being injured during a period (2006–2010) of intensive military deployment of Dutch troops under high-risk conditions. This may have its main limitation due to a reduction in accuracy when asking about participants’ recall.^55^ The mode of military operations, including the medical support chain, in the aforementioned period remained largely the same until the start of the war in Ukraine. However, service member’s expectations of what is important to them under current conditions and the continued development of (civilian) standards of care may need to reconsider preconditions for medical outcomes to the current military health system. Furthermore, the longer time between experiences and data collection may provide a lower mental health burden for the participants. We decided not to involve any partners or family of the group 2 participants. Involving family in the ‘WSM care pathway’ may influence the preconditions found and would still have to be examined.^56 57^ The use of the experienced expert panel was able to supplement this to some extent. Many of the preconditions used are formulated as a condition; however, some preconditions are formulated with a reference to the past and Uruzgan.

The use of the narrative style review method has provided broad insight into a field in which the provision of patient-centred care and medical outcomes is largely unexplored. In future research, a systematic review will need to be conducted to convert preconditions into validated quality indicators. The Delphi study reached consensus on a set of preconditions and provided guidance on the perceived importance of delivering patient-centred care and medical outcomes during military operations. However, in line with the limitation described above regarding quality indicators, the study does not provide definitive guidance. It is recommended that the findings of this study should be further tested through more robust research to improve the consistency and quality of the data.

### Conclusions

With this study, we have identified a consensus on the perceived importance of a set of preconditions that can contribute to the implementation of VBHC concept applications in a MHS. Currently, there are no references for measuring the desired relevant medical outcome of the WSM from the perspective of trauma care during operational deployment. The ICHOM Set for Patient-Centred Outcome Measures for Patients with Major Injury,^11^ together with the preconditions established by consensus in this study, can be used in future research as building blocks for the military application of VBHC. The use of the VBHC concept can contribute to the objective measure of creating value for the patient from the perspective of the care recipient him or herself, the care provider and the care facilitator. The result of this study can be used as a starting point for future research on the application of VBHC, improvement of health outcomes that matter to the patient and as a communication tool to improve relationships and mutual understanding between the military patient, military healthcare professional and MCO. Finally, measuring health outcomes can elucidate how individual readiness aligns with system readiness, underscoring that optimal health outcomes are an important precondition for military operational readiness.

## Supplementary material

10.1136/bmjopen-2025-101224 online supplemental table 1

10.1136/bmjopen-2025-101224 online supplemental table 2

10.1136/bmjopen-2025-101224 online supplemental table 3

10.1136/bmjopen-2025-101224 online supplemental table 4

## Data Availability

Data are available upon reasonable request. All data relevant to the study are included in the article or uploaded as supplementary information.
