# Supplementary material for: Development of a set of value-based healthcare preconditions supporting military trauma patients in military operations: a Delphi study
Source: BMJ Open. 2025 Dec 19;15(12):e101224. doi: 10.1136/bmjopen-2025-101224 (PMC12716574; doi:10.1136/bmjopen-2025-101224)
Supplement: online supplemental table 2 [file bmjopen-15-12-s002.pdf]

Supplementary material S2. Definitive list with 17 preconditions.

No.	Precondition
1	The conditions of repatriation are clear and must be respected unequivocally.
2	There is an 'informed consent' between patient and professional.
3	During the preparation (the mission preparation training) for the deployment, the 'procedure in the event of injury' was known in role 2 MTF in Uruzgan.
4	The treatment of the patient during deployment may not differ from the treatment in the Netherlands.
5	All of the wounded soldier’s wishes for treatment under special circumstances (treatment wishes) were stated in the medical file.
6	During the period of deployment at the R2 MTF, there were recognizable moments of consultation between the patient and the professional, to discuss the treatment process together, known as shared decision making (SDM).
7	The patient is involved in the time-out procedure at the OR.
8	By placing more emphasis on consultation and making agreements, by the actors in the medical chain, added value is created for the treatment of the patient and his/her outcome.
9	Sufficient information must be available when reporting injuries (NATO 9-liner[18]) to the R2 MTF.
10	Patient safety must not be compromised.
11	Delay of care in the deployment area must be kept to a minimum in view of its effect on medical outcomes.
12	There was a choice to adjust the time of departure back home (in the event of operational, medical and/or patient interests).
13	Based on the number of patients, it is possible to work event-driven, in which case the established procedures weren’t leading, but the circumstances regarding the wounded soldiers.
14	The registration of the treatment was carried out on time from entry to discharge, which led to a complete patient record.
15	Depending on the severity of the injury, the patient has the choice of where and when the treatment will take place.
16	An (operational) client council is necessary to represent and safeguard the interests of patients during operational deployment.
17	Depending on the severity of the injury, the military personnel's family must be explicitly involved in any treatment program.
