# Supplementary material for: Development of a set of value-based healthcare preconditions supporting military trauma patients in military operations: a Delphi study
Source: BMJ Open. 2025 Dec 19;15(12):e101224. doi: 10.1136/bmjopen-2025-101224 (PMC12716574; doi:10.1136/bmjopen-2025-101224)
Supplement: online supplemental table 3 [file bmjopen-15-12-s003.pdf]

Supplementary material S3. Overview comments of participants Delphi survey			Group 1		Group 2
**Item number**	**Title item**	**Comment (*participant quotes*)**	**MCO**	**MS**	**WSM**
1.	The conditions of repatriation are clear and must be respected unequivocally.				
		Dependent on function	0	1	0
		Depending on the clinical picture and recurrence rate	0	1	0
		Dependent on alloy and operational circumstances may make it necessary to deviate from "remain in deployment zone"	0	1	0
		Illness contracted must lead to deployment restriction	1	0	0
		Limit of 3 weeks is fine. Also depends on the circumstances, of course.	0	1	0
		Depends on the medical cause	0	1	0
		Add depending on operational conditions, function of soldier involved and possible remaining deployment time. Also insert: repatriation is a commander's decision.	1	0	0
		expected recovery within a week	0	1	0
2.	There is an 'informed consent' between patient and professional.				
		Depending on whether this possibility is there (depending on urgency and awareness)	1	0	0
		Record and sign everything in writing and know own competence and ability	0	1	0
		With foreign units/locals written consent	0	1	0
		this is extremely difficult when treating local patients	0	1	0
		in plain language	0	0	1
		It is usually an emergency procedure and does not apply	0	1	0
		Add: in patients who can react adequately	0	1	0
3.	During the preparation (the mission preparation training) for the deployment, the 'procedure in the event of injury' was known in role 2 MTF in Uruzgan.				
		If a patient wants to set limits on their treatment, it is important that they think about this beforehand.	0	1	0
		The wishes of the individual can sometimes be secondary to the collective depending on the operational context. Military personnel should also be told this. Despite the fact that for doctors everything often seems like an individual case, the individual and his (desired) treatment is part of the total readiness.	1	0	0
		ilv wishes for treatment mention the possibilities for treatment	0	1	0
		Is addressed ipv known	1	0	0
		is the question whether it is known, or is the question whether it is important?	1	0	0
		Not everything can be foreseen	0	1	0
		not enough attention was paid to it	0	0	1
		Note: I don't remember this point ever being addressed. I hadn't wanted to fill in anything.	1	0	0
4.	The treatment of the patient during deployment may not differ from the treatment in the Netherlands.				
		As a doctor you also need to know the different functions	0	1	0
		When treating a soldier during deployment, the same level of treatment is aimed for as in the Netherlands	0	1	0
		Deployment is often about survival and not about redeployment	0	1	0
		Where possible, if not possible, describe limitations in advance	0	1	0
		The wounded soldier on mission may expect good care (cf. articles 2 and 3 Smits), given the operational circumstances.	1	0	0
		Approaching the level of civilian healthcare is a correct and good aim, but cannot always lead to that level under operational circumstances.	1	0	0
		Should not differ in essence	0	1	0
		Note: Qualitatively different treatment almost by definition	1	0	0
		Add :as much as possible	0	1	0

5.	All of the wounded soldier’s wishes for treatment under special circumstances (treatment wishes) were stated in the medical file.				
		Depends on patient's mental state	0	1	0
		Treatment wishes are nice, but not necessarily feasible. In Care under Fire, the caregiver should not have to think about these wishes. Then just provide protocolised assistance. At Role 2 or above, there is room for this. In large-scale violence in a multi-domain hybrid environment, the wishes of the individual can also be pursued, but fulfilment is not always feasible either.	1	0	0
		Cfm thesis followed by , however, whether these wishes can be met depends on the circumstances.	1	0	0
		Possibly supplement the statement with "in the mission area" , additional wishes in the Role4 may be met.	1	0	0
		Donation procedure is not possible. Other restrictions will not be addressed until later	0	1	0
		People with clearly different wishes may not need to be deployed	0	1	0
		NB THIS IS ABOUT INTERNATIONAL.	0	1	0
		not clearly mapped out beforehand	0	0	1
6.	During the period of deployment at the R2 MTF, there were recognizable moments of consultation between the patient and the professional, to discuss the treatment process together, known as shared decision making (SDM).				
		If possible, then fine. I have also seen examples, where there was consultation time enough but they did not reach a shared decision. That is also always possible.	1	0	0
		but not implemented during uruzgan	0	1	0
		not always applicable, often a patient is not conscious	0	0	1
		Comment: have there been any? Wonder.	1	0	0
		Often they are urgent cases and there is no time for this. If not, it can be discussed further in the home country.	0	1	0
7.	The patient is involved in the time-out procedure at the OR.				
		Stressing no difference with civilian interventions	0	1	0
		Is rarely, if ever, possible in damage control	0	1	0
		Patient can also contribute, to the extent possible, on matters affecting him.	0	1	0
		often not applicable, unconscious	0	0	1
		add as far as possible	0	1	0
8.	By placing more emphasis on consultation and making agreements, by the actors in the medical chain, added value is created for the treatment of the patient and his/her outcome.				
		Really nice story in a peaceful environment that is not under pressure (high supply, operational conditions, low stocks, etc). This sounds like fine peacekeeping, or low intensity war fighting. Pursuit, even in high-intensity some fighting, can't hurt. Of course, the aim of operational health care is to get or keep soldiers fighting again.	1	0	0
		In the mission area? Only deployment-ready soldiers are deployed. DNBIs threatening to last longer than 21 days are subject to repatriation rules.	1	0	0
		Explanation could be clearer. Does it relate to the chain or is it about Role 2	1	0	0
		Especially in the context of repatriation of severely wounded.	0	1	0
		As discussed in the preparation phase	0	1	0

9.	Sufficient information must be available when reporting injuries (NATO 9-liner[18]) to the R2 MTF.				
		Using an example life liner	0	1	0
		The 9-liner is primarily intended as an application for MEDEVAC. MIST is added as additional information. Numbers, priority and MIST are used to choose the appropriate treatment capacity. In practice, prioritisation and MIST are often found to be incomplete/incorrect. Therefore, worst case is assumed.	1	0	0
		Contributes to adequate care of the injured	0	1	0
		Actually only number of patients important	0	1	0
		Very important, but medical teams should also be trained to treat as adequately as possible with the least amount of information. During combat conditions, information may be incomplete, corrupt or absent	1	0	0
		correctly link einfo to home front	0	0	1
		maximum information ilv sufficient	0	1	0
		As long as this does not lead to delays!	0	1	0
10.	Patient safety must not be compromised.				
		Earlier than in NL, it may come to fierce triage decisions	0	1	0
		Agreed	1	0	0
		Should be agreed well in advance. In emergencies, this is no longer possible.	0	1	0
		Statement can only have one answer, I think. Can't imagine anyone not considering it important.	1	0	0
11.	Delay of care in the deployment area must be kept to a minimum in view of its effect on medical outcomes.				
		Depending on the severity of the injury, appropriate treatment should be provided as soon as possible	0	1	0
		This is a fundamental principle. But it is not always in your control.	1	0	0
		and again put in writing and sign	0	1	0
		After outcomes insert "and given the circumstances"	1	0	0
12.	There was a choice to adjust the time of departure back home (in the event of operational, medical and/or patient interests).				
		Surgeons will often be unaware of the options	1	0	0
		How there was an option	0	1	0
		I don't understand the statement	0	1	0
		Operational importance also counts. The military should know that beforehand.	0	1	0
		fine	0	1	0
13.	Based on the number of patients, it is possible to work event-driven, in which case the established procedures weren't leading, but the circumstances regarding the wounded soldiers.				
		'Event-driven' should just be one of the standard procedures. How is this excluded from established procedures?	1	0	0
		A deployment/war zone is different from a home situation and different criteria may apply.	0	1	0
		Event Driven are not only based on patient supply (MASCAL) but also by operational conditions (bunker alarm, CBRN).	1	0	0
		Should have been discussed in the preparation phase as well	0	1	0
		Opm: event driven and following procedures go together just fine.	1	0	0
		What do you mean by at the front	1	0	0
		follow procedures as much as possible	0	1	0
14.	The registration of the treatment was carried out on time from entry to discharge, which led to a complete patient record.				
		ABCD protocol takes precedence over completing a status	0	1	0
		Complete record requires more than just correct times	0	1	0
		This is both in the interest of the patient and also to evaluate the medical effect.	0	1	0
		There is still insufficient opportunity for IDR staff to gain insight into the patient record (GIDS)	0	1	0
		None. The practice was different	0	1	0

15.	Depending on the severity of the injury, the patient has the choice of where and when the treatment will take place.				
		If circumstances permit	1	0	0
		In accordance with health insurance agreement, this is not true	0	1	0
		The wish can be expressed, but is not always feasible	0	1	0
		That choice option will rarely be there	0	1	0
		Do no further harm	0	1	0
		possibly also consult with the home front	0	0	1
		Said example about whether or not to amputate can lead to serious harm to the pt. In such cases, the dd surgeon has to apply emergency treatment according to the rules, almost all pt will say "do not amputate" which may put them at risk.	0	1	0
		the team decides	0	1	0
		But medically advised	0	1	0
		Circumstances are not mentioned is there time and space to choose?	1	0	0
		Often there is no such choice because there is a closed health system. Timeliness, distance and location somewhere in the world can greatly limit choice.	1	0	0
16.	An (operational) client council is necessary to represent and safeguard the interests of patients during operational deployment.				
		Make negotiable in preparation phase	0	1	0
		civilian yes operationally no	0	1	0
		I see this more as an advisory board in the Netherlands. In a deployment area, this is difficult.	0	1	0
		I think this is overkill if you want this to apply to the operational setting as well. Perhaps in a long-term low-intensity mission this could be done, but who has time for this?	1	0	0
		Is not comparable to NL	0	1	0
		Not during deployment but fix things in advance	0	1	0
		Replace necessary with "contributes to"	1	0	0
17.	Depending on the severity of the injury, the military personnel's family must be explicitly involved in any treatment program.				
		depending on what the military wants	0	1	0
		If it can be done, this is nice.	1	0	0
		Limited applicability.	1	0	0
		Do no further harm	0	1	0
		Family cannot oversee eea remotely. It is then about treatments and repatriation. In such cases, the pt himself is the only one who can co-decide.	0	1	0
		Insert "at the request or with the consent of the wounded soldier".	1	0	0
		After return	0	1	0
		not realistic and not possible due to distance	0	1	0
		As far as the situation allows.	0	1	0
	**Number comments: total / per (sub)group**	**115**	**37**	**71**	**7**
	**Percentages**		**32%**	**62%**	**6%**
