# Supplementary material for: Development of a set of value-based healthcare preconditions supporting military trauma patients in military operations: a Delphi study
Source: BMJ Open. 2025 Dec 19;15(12):e101224. doi: 10.1136/bmjopen-2025-101224 (PMC12716574; doi:10.1136/bmjopen-2025-101224)
Supplement: online supplemental table 4 [file bmjopen-15-12-s004.pdf]

Supplementary material S4 – Clarification of items after Delphi survey

	No.	Included statement	Clarification
Highest perceived importance	2.	There is an 'informed consent' between patient and professional[45].	The legal basis for informed consent is laid down in the Dutch Medical Treatment Contracts Act (Dutch: WGBO), also for informed consent in the emergency setting[46]. Both groups indicate that this is important. Professionals and one commander comment that the patient must be able to respond. This is not necessary, but there is an explicit obligation to provide information. And as much as possible in understandable text for everyone.
	10.	Patient safety must not be compromised.	Patient safety is described in the literature[47], the definition has been added to the statement and is considered important by almost everyone. The statement is very important for treatment in acute care to properly regulate the care around the patient.
	14.	The registration of the treatment was carried out on time from entry to discharge, which led to a complete patient record.	Patient registration has been established in the Netherlands; this has also been unanimously judged to be of minimal importance for the mission area. Comments were mainly focused on working on the patient record in case of time pressure. This can be taken into account when establishing the statement which leads to an indicator for good care[10]. This underlines the importance of this statement for acute care in the mission area.
High perceived importance	7.	The patient is involved in the time-out procedure at the OR.	The time-out procedures are described in the literature as a set control moment to start the surgical procedure[50]. However, one surgeon believes that this is 'somewhat' important. This is reflected in the comments on the statement, as it is indicated that the time out is not always possible with Damage Control Surgery (DCS). This statement is included and is therefore viewed as important in acute care, with perhaps the caveat discussed above. the 'somewhat' importance can be supported by a group 2 comment that the injured service member may also be unresponsive.
	3.	During the preparation (the mission preparation training) for the deployment, the 'procedure in the event of injury' was known in role 2 MTF in Uruzgan.	The preparation for a deployment does not provide any information on 'procedure getting wounded'. Nevertheless, all respondents consider this to be important or very important, and it will have to be included in a good preparation for deployment[52].

Perceived importance	6.	During the period of deployment at the R2 MTF, there were recognisable moments of consultation between the patient and the professional to discuss the treatment process together, known as shared decision making (SDM).	In the healthcare domain, it is common practice to have consultation between the professional and the patient, SDM[27]. It is remarkable that the 'not directly involved' group of commanders considers this more important than the professionals. Of the professionals, 75% indicate that it is minimally important and 100% of the commanders say it is important. SDM has not been formalised but, in view of the outcome, it is certainly important when treating patients in acute care. However, a group 2 comment emphasized on to consider that an injured service member may also be unresponsive.
	8.	By placing more emphasis on consultation and making agreements, by the actors in the medical chain, added value is created for the treatment of the patient and his/her outcome.	This is a process agreement in the military medical chain[4] that is assessed as important or very important by the professionals. One commander does not consider it important because, in his opinion, military personnel should always be ready for deployment. It is possible that the statement has been interpreted differently. The professionals all believe that it is important or very important for the patient.
	9.	Sufficient information must be available when reporting injuries (NATO 9-liner[20]) to the R2 MTF.	The notification is formally laid down in NATO doctrine[54]. In the Netherlands, a patient is also announced before arrival by the person/unit sending the patient in. One professional and one commander find it somewhat important; the others find it important or very important. This statement is important for the (acute) chain in which the service members work. One wounded service member also argued for sufficient information, which could inform the home front in a better way.
Opposing ranking in perceived importance by group 1 & 2	11.	Delay of care in the deployment area must be kept to a minimum in view of its effect on medical outcomes.	Delay of care is described by everyone as being minimally important, and the literature also emphasises that speed of action is very important in acute care[32]. It is also true that treatment must be pursued in the right place and at the right time[44]. In this statement, this is therefore unanimously endorsed as (very) important for acute care.
	13.	Based on the number of patients, it is possible to work event-driven, in which case the established procedures weren't leading, but the circumstances regarding the wounded soldiers.	This statement is based on the experiences of the expert panel, where event-driven events are guided by circumstances rather than procedure [48]. This resulted in a differentiated outcome between the professionals, unfortunately the professionals who did not consider this important did not comment on this statement, meaning we do not know the reasons behind their choices. Many of the target groups believed that it was minimally important; this was the decisive factor in valuing the statement as important. This statement was not immediately assessed as important in the first round, but it was assessed in such a way that the statement was taken to the second Delphi round where it was included.

Additional perceived importance by wounded service members (group 1)	*5.*	*All of the wounded soldier's wishes for treatment under special circumstances (treatment wishes) were stated in the medical file.*	*Group 2 found this item important, especially focusing on honouring the service member his/her wishes. It was also noted that this topic is not discussed in preparation and during the deployment. Group 1 excluded this item because it is certainly something to be respected but often not possible, especially not donor-related actions. However, it was indicated that when possible, in Role 2, but certainly Role 4, implementation of wishes could be looked at.*
	17.	*Depending on the severity of the injury, the military personnel's family must be explicitly involved in any treatment program.*	*Group 2 found this item very important. This is also more in line with how healthcare is conducted today. People also understood the special circumstances and limitations during deployment. Group 1 excluded this item, also substantiated by the aforementioned special circumstances and limitations during deployment. However, it was indicated that when possible and at the wish of the injured service member, this should certainly be possible.*
