# Supplementary material for: Development of a set of value-based healthcare preconditions supporting military trauma patients in military operations: a Delphi study
Source: BMJ Open. 2025 Dec 19;15(12):e101224. doi: 10.1136/bmjopen-2025-101224 (PMC12716574; doi:10.1136/bmjopen-2025-101224)
Supplement: Supplementary file 5 [file bmjopen-2025-101224.reviewer_comments.pdf]

PEER REVIEW HISTORY

BMJ Open publishes all reviews undertaken for accepted manuscripts. Reviewers are asked to complete a checklist review form and are provided with free text boxes to elaborate on their assessment. These free text comments are reproduced below.

ARTICLE DETAILS

Title (Provisional)

Development of a set of value-based health care preconditions supporting military trauma patients in military operations: a Delphi study

Authors

van der Wal, Henk; Dulk, Tonnie; Dongen, van, Thijs; Hoencamp, Rigo; Ahaus, Kees

---

VERSION 1 - REVIEW

**Reviewer**	**1**
**Name**	**Dilday , Joshua**
**Affiliation**	**Medical College of Wisconsin**
**Date**	**18-Apr-2025**
**COI**	**None**

This manuscript describes the implementation of a Delphi model to determine consensus on necessary preconditions for incorporating value-based healthcare models in military traumatic situations. The authors compared results between the two groups and identified thematic areas of overlap and contrast. While there were expected differences in some thematic elements between the groups, the results established a ranking of similarly valued conditions affecting patient care on the battlefield. I commend the authors for undertaking the task of developing a framework for incorporating patient-centered and value-based criteria in a deployed environment. After reading this paper, I am left with a few questions and comments for the authors.

1. The introduction seems a bit long. Shortening it could help highlight the aim of exploring VBHC models in to military trauma better. As it stands, it seems the VBHC portion gets lost within the NATO doctrine background.

2. Overall, the discussion highlights the key points of the Delphi survey. However, it still seems too long. While the separation of the key tenets is appreciated, I believe each point can be shortened to avoid losing the overall message of each point. One suggestion might be to house some of the discussion points in the results section, as they seem to describe the results of the survey without significant additional commentary.

3. The authors mention the limitations due to the military climate in Afghanistan compared to Ukraine. While the wounded perspective might not change, do the authors anticipate any differences from the commander/surgeon perspective in the changing conflict environments seen with the Ukraine-Russia conflict?

4. Why lump surgeons with commanders? Sometimes those decision-making situations are different. Is there any thought that the agreed-upon criteria might be different if three groups were compared (surgeon vs. commander vs. wounded)?

**Reviewer**	**2**
**Name**	**Berube, Melanie**
**Affiliation**	**CHU de Quebec-Universite Laval**
**Date**	**05-Oct-2025**
**COI**	**None**

Review

Development of a set of value-based health care preconditions supporting military trauma patients in military operations: a Delphi study

General comments:

• Please spell out the abbreviations the first time they are used throughout the manuscript.

• Tables: The same number of decimals should be used. Also, dot should be used prior to decimals instead than comma.

• Abbreviations used in Tables and Figures, should be presented and spelled out at the bottom of them.

Abstract:

• They are two outcomes presented (military-oriented preconditions and patient centred outcome measures), but only results related to preconditions are presented.

Introduction:

• Please reformulate this sentence: Readiness of the individual service member…can be seen as a precondition for readiness…

• The definition of Value-Based Healthcare is not entirely accurate. It is missing some important elements: The central tenant of the VBCH is that healthcare delivery must be organized around the patient’s needs, accurately measure the outcomes that matter to patients, and measure the costs to achieve them.

• The meaning of “preconditions” should be explained.

• Reference is made to the work of the Consortium for Health Outcomes Measurement. How does the work presented in this manuscript differ?

Methods:

• A Delphi study is based on the involvement of experts. The section on the expert panel is confusing because it appears to have been involved before the Delphi study. The content of this section should be moved to the section explaining how the Delphi questionnaire was developed.

• A narrative review is not a valid method for identifying quality indicators. Recognizing that the work is based on this narrative review, the authors should provide more details on the methodology used in this narrative review and also add details on the subject in the limitations section.

• A 4-point Likert scale is not sufficiently discriminating. The authors should provide a reference supporting the use of this type of scale by linking it to the Delphi method used.

• Please provide more details on this: The SD (standard deviation) of a prerequisite was mainly used to rank prerequisites with a similar average. How was the SD used in practice to establish the ranking?

Results:

• The following sentences should be moved in the methods section: Before approaching the participants, we looked for a balance between participants providing and facilitating care (Group 1) and those receiving care (Group 2); In addition to the balance of background, we also assessed at the appropriate experience composition among the two groups.

• Why did group 1 have to decide on 9 predictions and group 2 on 7 of them? This should be explained in the method.

• Preconditions added from round 1 and 2 should be presented or the authors should refer to the related tables where they are presented in the manuscript.

• Table 4: The preconditions should be reworded to make it clear what must be done (must be, should be, etc.). Some predictions are worded this way, while others are not.

• The preconditions deemed most important by both groups should be presented in the text and not only in the tables.

• The following sentence is incomplete: Next, it was noticed that between the two groups, precondition 11 ‘Delay of care in the deployment area must be kept to a minimum in view of its effect on medical outcomes.’ and precondition 13 ‘Based on the number of patients, it is possible to work event-driven.

• The following is not a result, but an interpretation of findings and should therefore be moved in the discussion section: In which case the established procedures weren’t leading,

but the circumstances regarding the wounded soldiers.' were scored as absolutely most important within the respective group, contrasting organisational versus patient.

Discussion:

• The discussion should summarize the main findings of the study and then contrast these findings with other studies on the subject or existing guidelines. It is also important to explain in the results section the differences obtained between the two groups and how these differences should be taken into account when implementing the identified preconditions.

Conclusions:

• The following sentence contains several pleonasms and needs to be rewritten: Finally, measurement of health outcomes may contribute to the insight of an individual's readiness, which could provide insight into the readiness of a military health system as a whole.

VERSION 1 - AUTHOR RESPONSE

Reviewer: 1

Dr. Joshua Dilday , Medical College of Wisconsin

Comments to the Author:

This manuscript describes the implementation of a Delphi model to determine consensus on necessary preconditions for incorporating value-based healthcare models in military traumatic situations. The authors compared results between the two groups and identified thematic areas of overlap and contrast. While there were expected differences in some thematic elements between the groups, the results established a ranking of similarly valued conditions affecting patient care on the battlefield. I commend the authors for undertaking the task of developing a framework for incorporating patient-centered and value-based criteria in a deployed environment. After reading this paper, I am left with a few questions and comments for the authors.

Author response: Thank you for your time and effort to review our manuscript. We have studied your comments and have addressed them in this document and in the revised manuscript.

1. The introduction seems a bit long. Shortening it could help highlight the aim of exploring VBHC models in to military trauma better. As it stands, it seems the VBHC portion gets lost within the NATO doctrine background.

Author response: Thank you for this observation. We have shortened the introduction section, to emphasize more on the VBHC aspects than on the military aspects.

2. Overall, the discussion highlights the key points of the Delphi survey. However, it still seems too long. While the separation of the key tenets is appreciated, I believe each point can be shortened to avoid losing the overall message of each point. One suggestion might be to house some of the

discussion points in the results section, as they seem to describe the results of the survey without significant additional commentary.
Author response: Thank you for the comment, we agree with you that the discussion section is too long. In particular, we shortened the paragraph entitled ‘target group observations’ by rewriting part of the paragraph and transferring the data elements to the results section. This concerns both the number of suggestions and what they relate to. We also rewrote the relevant paragraph in the results section to make it more concise. We also reduced the importance of included preconditions section. We feel the Discussion section is now more in balance.

3. The authors mention the limitations due to the military climate in Afghanistan compared to Ukraine. While the wounded perspective might not change, do the authors anticipate any differences from the commander/surgeon perspective in the changing conflict environments seen with the Ukraine-Russia conflict?
Author response: Thank you for your observation on this important point. We have decided to certainly mention this as a limitation, but from a broader perspective. This is supported by two points. Firstly, our data collection took place in the period 2020/2022, and we want to remain close to the findings from that period in our research. Secondly, changes in the conflict environments can be seen, but mainly from a clinical perspective. In our opinion, the objective of this research, namely patient-centred care and health outcomes, is currently still underexplored.

4. Why lump surgeons with commanders? Sometimes those decision-making situations are different. Is there any thought that the agreed-upon criteria might be different if three groups were compared (surgeon vs. commander vs. wounded)?
Thank you for this valuable comment. It is acknowledged that a distinction must be made between the perspectives of military surgeons and commanders. However, the objective of this study was to explore and compare two overarching perspectives: a clinical/patient-centred perspective and a logistical/operational perspective. The first of these groups is represented by military surgeons, while the second is represented by commanding officers. In order to ensure sufficient analytical power and balanced group sizes, these two professional roles were combined into one group (n = 17), which was then compared with the wounded service members (n = 13). This methodological approach facilitated the establishment of a contrast between the clinical-operational perspective and that of the patient, thus aligning with the study's VBHC-oriented objective of understanding both the provider and patient viewpoints. In subsequent follow-up studies conducted by the first author, these subgroups have been examined separately to explore their distinct roles and the applicability of VBHC principles in greater detail.

Reviewer: 2
Prof. Melanie Berube, CHU de Quebec-Universite Laval, Universite Laval
Comments to the Author:
Review

Development of a set of value-based health care preconditions supporting military trauma patients in military operations: a Delphi study

Author response: Thank you for your detailed feedback and effort to review our manuscript. We have addressed your comments in this document and in the revised manuscript.

General comments:

• Please spell out the abbreviations the first time they are used throughout the manuscript.

Author response: Thank you for this observation, we have spelled out the following abbreviations the first time throughout the manuscript: ACCORD = Accurate Consensus Reporting Document; ICHOM = International Consortium for Health Outcomes Measurement; MCO = Military Commanding Officer; MEC = Medical Ethical Commission; MHS = Military Health System; MS = Military Surgeon; MTF = Medical Treatment Facility; NATO = North Atlantic Treaty Organization; OR = Operating Room; SD = Standard Deviation; SDM = Shared Decision-Making ; VBHC = Value-Based Healthcare; WIA = Wounded in Action ; WSM = Wounded Service Member

• Tables: The same number of decimals should be used. Also, dot should be used prior to decimals instead than comma.

Author response: Thank you for reading so precisely, we have changed to the same number of decimals by using dot, instead of comma.

• Abbreviations used in Tables and Figures, should be presented and spelled out at the bottom of them.

Author response: Thank you for your observation, we have spelled out all abbreviations at the bottom of all tables.

Abstract:

• They are two outcomes presented (military-oriented preconditions and patient centred outcome measures), but only results related to preconditions are presented.

Author response: Thank you for this comment. We have amended the outcome measures section of the abstract, focusing now more on the preconditions as described in the results paragraph. The second outcome now focuses on a conceptual contribution to future developments in patient-centred outcome measures. It reads as follows: '*Secondary outcome: conceptual contribution to the future development of patient-centred outcome measures for military trauma populations.'*

Introduction:

• Please reformulate this sentence: Readiness of the individual service member…can be seen as a precondition for readiness…

Author response: Thank you for your feedback, we have reformulated the sentence to also relate better to the aforementioned text in the paragraph. It now reads as: '*Although doctrine defines it as patient-centred, its operational reality remains largely output-oriented - focused on readiness, throughput, and treatment capacity - rather than outcome-oriented, prioritising long-term recovery, functionality, and quality of life. Nonetheless, there is a clear relationship between the health*

outcomes of individual service members and the readiness of the medical system. ***Contributing to good health outcomes can therefore be seen as a precondition of the medical system for military operational readiness.'***. The last sentence of this text (in bold) is the result of the reformulation of the sentence and also the reformulation of the Introduction section.

• The definition of Value-Based Healthcare is not entirely accurate. It is missing some important elements: The central tenant of the VBHC is that healthcare delivery must be organized around the patient's needs, accurately measure the outcomes that matter to patients, and measure the costs to achieve them.

Author response: Thank you for the feedback on the need to address more on the concept of VBHC. We have reformulated the text around the definition on VBHC, also better connecting with the subsequent text in the paragraph. It reads as follows: VBHC defines value as the achieved health outcomes that matter most to patients relative to the costs required to achieve them advocating a transformation from volume-based to outcome-based healthcare. Its implementation requires organising delivery of care around patient needs, measuring outcomes across the full cycle of care, and using these data to continuously improve quality and efficiency.

• The meaning of "preconditions" should be explained.

Author response: Thank you for addressing this omission, which must be resolved indeed. We have added a definition and a short clarification on preconditions in the last paragraph of the Introduction section. Both read as: '*Our goal was to identify preconditions -* ***defined as the essential medical, logistical, operational, and contextual factors that must be in place to enable the delivery of patient-centred care and value-based healthcare within the military operational environment*** *- for the preferred and relevant medical outcomes for military patients with acute trauma during military operations. In this* ***context****, preconditions encompass the balance between what is clinically feasible (as determined by healthcare professionals following medical guidelines and available resources) and what is logistically and operationally possible (as determined by military leadership). These elements represent the fundamental requirements that determine the potential for achieving desired medical outcomes and patient-centred approaches during military operations*.' In bold the definition and after the word context in bold the clarification.

• Reference is made to the work of the Consortium for Health Outcomes Measurement. How does the work presented in this manuscript differ?

Author response: Thank you for your comment and the difference was not very clear before a better explanation of our use of preconditions. The difference from ICHOM focusing on solely outcome measurement and our use of preconditions is that we have addressed not only outcomes and outcome measurement but also logistical and operational feasibility.

Methods:

• A Delphi study is based on the involvement of experts. The section on the expert panel is confusing because it appears to have been involved before the Delphi study. The content of this section should be moved to the section explaining how the Delphi questionnaire was developed.

Author response: Thank you for your comment. We have the biggest part of the expert panel part in the Methods moved to the Delphi method part, which makes it more clear that the expert panel was involved in the precondition development but was not part of the Delphi study process.

• A narrative review is not a valid method for identifying quality indicators. Recognizing that the work is based on this narrative review, the authors should provide more details on the methodology used in this narrative review and also add details on the subject in the limitations section.

Author response: Thank you for your observation. In light of the research objective to identify preconditions in an under-researched field that lacks standardised terminology, a narrative literature review was deemed a more appropriate approach than strictly validated evidence. We therefore proceeded with a modified Delphi method (Vleugelers et al., 2020), which, in contrast to a conventional Delphi approach that allows experts considerable autonomy, utilises a literature-based approach to minimise the time and effort required. We also addressed the used of the narrative review in the limitations section.

• A 4-point Likert scale is not sufficiently discriminating. The authors should provide a reference supporting the use of this type of scale by linking it to the Delphi method used.

Author response: Thank you for your feedback on this aspect of using a 4-point Likert scale. Based on existing literature (e.g., Wu et al., 2024) we decided to use a 4-point Likert scale to get stable findings for our Delphi questionnaire. We have provided references to support this type of use (Akins et al., 2005; Vogel et al., 2019). We also added some text at the limitations section to emphasize that more robust future research is needed to get definitive guidance.

• Please provide more details on this: The SD (standard deviation) of a prerequisite was mainly used to rank prerequisites with a similar average. How was the SD used in practice to establish the ranking?

Author response: Thank you for your feedback. When there was a similar mean the SD was used as a secondary ranking criterion, with the lowest SD indicating more consistent ratings, showing stronger agreement. We have explained this in the text under ranking of the preconditions.

Results:

• The following sentences should be moved in the methods section: Before approaching the participants, we looked for a balance between participants providing and facilitating care (Group 1) and those receiving care (Group 2); In addition to the balance of background, we also assessed at the appropriate experience composition among the two groups.

Author response: Thank you for your observation. We have moved the sentences to methods section and updated the text in the beginning of the results section.

• Why did group 1 have to decide on 9 predictions and group 2 on 7 of them? This should be explained in the method.

Author response: Thank you for your comment. In the methods section we have explained the system of inclusion and exclusion including the criteria. In table 3 Group 1 had 9 of 17 preconditions included, and they had to vote again on the remaining 6 which were inconclusive. Group 2 had 7 of 17 preconditions included and had to vote again on the remaining 9 inconclusive. This shows the

progress on the 2 Delphi rounds we conducted.

• Preconditions added from round 1 and 2 should be presented or the authors should refer to the related tables where they are presented in the manuscript.

Author response: Thank you for your observation. We have made this clear by adding more clarification in the results section, referring to Tables 4 and 5, and explicitly mentioning the definitive excluded preconditions.

• Table 4: The preconditions should be reworded to make it clear what must be done (must be, should be, etc.). Some predictions are worded this way, while others are not.

Author response: Thank you for this valuable observation. We agree that the preconditions are inherently normative in nature and should be interpreted as statements indicating what *must* or *should* be in place to enable patient-centred and value-based trauma care. During the Delphi process, all statements were presented to participants explicitly as *preconditions*—that is, as requirements considered necessary for achieving the desired quality and outcomes of care. For this reason, we have not reworded individual items (e.g. by adding "must be" or "should be"), to avoid altering the phrasing of validated Delphi statements. Instead, we have added to the Methods and Results sections that all preconditions should be interpreted as normative statements.

• The preconditions deemed most important by both groups should be presented in the text and not only in the tables.

Author response: Thank you for your comment, we have updated the text to present the preconditions in full in the main text of the manuscript.

• The following sentence is incomplete: Next, it was noticed that between the two groups, precondition 11 'Delay of care in the deployment area must be kept to a minimum in view of its effect on medical outcomes.' and precondition 13 'Based on the number of patients, it is possible to work event-driven.

Author response: Thank you for this observation, and the text in the manuscript was correct, but the precondition 13 exist of 2 sentences, which caused the confusion. We have changed the dot to a comma and now it is a one sentence precondition with the exact same text.

• The following is not a result, but an interpretation of findings and should therefore be moved in the discussion section: In which case the established procedures weren't leading, but the circumstances regarding the wounded soldiers.' were scored as absolutely most important within the respective group, contrasting organisational versus patient.

Author response: Thank you for your remark and we have replaced this part to the discussion section. This is indeed a better place for the observation on those preconditions and connects better with the aim of that subsection.

Discussion:

• The discussion should summarize the main findings of the study and then contrast these findings with other studies on the subject or existing guidelines. It is also important to explain in the results section the differences obtained between the two groups and how these differences should be taken into account when implementing the identified preconditions.

Author response: Thank you for your observation, we have updated the discussion section to address this. We have put more emphasis on the general difference between both groups, by inserting also a comparable civilian study showing that there is more alignment than differences between patient and providers.

Conclusions:

• The following sentence contains several pleonasms and needs to be rewritten: Finally, measurement of health outcomes may contribute to the insight of an individual's readiness, which could provide insight into the readiness of a military health system as a whole.

Author response: Thank you for your comment, it now reads as a more concise sentence and a final concluding statement of the manuscript.

VERSION 2 - REVIEW

**Reviewer**	**1**
**Name**	**Dilday , Joshua**
**Affiliation**	**Medical College of Wisconsin**
**Date**	**20-Nov-2025**
**COI**	

This revised manuscript describes the implementation of a Delphi model to determine consensus on necessary preconditions for incorporating value-based healthcare models in military traumatic situations. The authors compared results between the two groups and identified thematic areas of overlap and contrast. While there were expected differences in some thematic elements between the groups, the results established a ranking of similarly valued conditions affecting patient care on the battlefield. I commend the authors for undertaking the task of developing a framework for incorporating patient-centered and value-based criteria in a deployed environment, and or addressing the previous questions/comments.
